# Standardizing norms for 180 coloured Snodgrass and Vanderwart pictures in Kannada language

**DOI:** 10.1371/journal.pone.0266359

**Published:** 2022-04-05

**Authors:** Shrilekha Bangalore, Holly Robson, Arlene J. Astell

**Affiliations:** 1 School of Psychology and Clinical Language Sciences, University of Reading, Reading, United Kingdom; 2 Department of Psychology and Language Sciences, University College London, London, England; 3 KITE, University Health Network, Toronto, Canada; 4 Departments of Occupational Sciences & Occupational Therapy and Psychiatry, University of Toronto, Toronto, Canada; Universita degli Studi di Milano-Bicocca, ITALY

## Abstract

This study presents normative data in Kannada for 180 coloured Snodgrass & Vanderwart pictures. Data are presented for naming latency, image agreement, picture-name agreement, familiarity, visual complexity, and age of acquisition (AoA). Sixty-eight native Kannada speaking adults completed all tasks. The effects of the rated variables on naming latency were examined and compared with data on the same variables in other languages. A regression analysis revealed that image agreement, name agreement, familiarity, and age of acquisition all had a significant impact on naming latency, while visual complexity and frequency did not. Although, the correlations among rated variables in Kannada were equivalent to previous normative studies, the cross-linguistic comparison revealed that only AoA was strongly correlated with other studies. The findings point to the importance of understanding the interplay of psycholinguistic variables on naming latency in different languages.

## Introduction

Picture naming is widely used in speech production research to address psycholinguistic, neuropsychological, and bi/multilingualism questions [1]. One of the most widely used sets of object-naming stimuli were produced by Snodgrass and Vanderwart [2]. Their set comprises 260-line drawings with norms for four psycholinguistic variables shown to operate during picture naming provided by a total of 219 American English speakers. Snodgrass and Vanderwart [2] split their participants into groups to collect ratings on the four psycholinguistic variables: image agreement (N = 42), name agreement (N = 40), familiarity (N = 40), and visual complexity (N = 40). The other 57 participants completed two additional image agreement tasks: Picture-Name agreement task (N = 40) and Image Variability rating (N = 17). These variables have been extensively investigated in multiple subsequent studies, with a colourized version of the images produced in 2004 [3].

Image agreement (IA) refers to the extent to which a given picture resembles an individual’s visual mental representation of a given object. It plays a key role [4–6] in accessing the perceptual representation of objects [7] whereby picture stimuli with high image agreement are named faster than those with low image agreement [4, 8–11]. Name agreement (NA) describes how much people agree on the dominant name for a pictured item. Many researchers, including Snodgrass and Vanderwart [2], calculate the information statistic *H* to examine name agreement. *H* captures information on the number of different names given for a picture by the participants and is calculated using the formula [12]

$$H=\sum_{i=1}^{K}\rho i\;log_{₂}\;\text{(}\frac{1}{\rho i}\text{)},$$

used by [2, 10]

where *k* refers to participants who gave an alternate name for an item. An item that achieves an *H* value of .0 has perfect name agreement, i.e., every participant gave the same name. Snodgrass & Vanderwart (1980) found 21% of their 260 items achieved a score of .0. NA is a strong predictor of latency and accuracy of word retrieval and comprehension in typical and clinical populations [5, 13–15]. Specifically, the names of pictures with high name agreement are comprehended and retrieved faster than those with low name agreement [9, 10].

Familiarity (FAM) is defined as an individual’s acquaintance with the pictured object and to what degree they encounter or think of the object in their everyday life. More FAM concepts are named quicker and categorized faster than unfamiliar and low familiarity items [14, 16–18]. FAM effects are known to be reliable but vary across studies with healthy adults [9] because the influence of FAM on naming latency are not consistent [4, 8]. The absence of FAM effects in naming picture, suggests object identification is not sensitive to frequency of occurrence [8]. Visual complexity (VC) refers to the amount of detail present within an image, such as the way lines are drawn, shape and colour (where coloured images are used). The intricacy of an image influences the picture naming speed such that images with more (sufficient) relevant details are easier to identify and are named faster compared to images with fewer (insufficient) distinguishing features [8, 9].

In addition to these commonly reported variables, several other psycholinguistic factors have been shown to influence performance on picture naming paradigms including word frequency and age of acquisition [4–6]. Age of acquisition (AOA) refers to the age at which the words are learned. Typically, this is achieved by asking the adults to rate the age they estimate they learnt the word according to age range provided [4, 19, 20]. The influence of AOA on lexical and semantic processing tasks (e.g, word naming, picture naming and lexical decision) includes faster and more accurate responses for the words and concepts acquired earlier in life [10, 19, 21]. Finally, word frequency is a measure of how often a word is used, generally based on the written or spoken corpora obtained through objective databases such as Francis & Kucera [22], Celex [23], rather than rating scales. In general, naming latency decreases as frequency increases [4, 9, 14, 20].

Whilst these findings have been widely reported, there is growing recognition of limitations of generalizing norms across languages [24]. First, cultural differences may result in some items being unknown in some parts of the world. For example, artichoke/asparagus/celery/chisel/nut/pliers/plug/raccoon/wagon/wrench/French horn/ were not known in Russian [15]. Other items may have very different frequency of occurrence in different languages such as harp/accordion/trumpet/flute which are included in English norms but have low familiarity in Turkish [20] for example. Second, images of items with low familiarity in any given culture may be perceived differently: e.g., /cigarette/ may be perceived as /pencil/. Third, some items may not have a specific name in a language and are known by their English names across cultures such as /penguin/, /stapler/ etc. Fourth, relationships between conceptual representations and lexical ones differ across languages, and an image which evokes a specific and single name in English might generate a more general name or multiple names in another language. For example, /bird/ is referred to as /pakshi/ or /hakki/ in Kannada, a Dravidian language spoken by at least 44 million [25] people in Karnataka in the Southwest of India, as well as linguistic minorities in Maharashtra, Andhra Pradesh, Tamil Nadu, Telangana, Kerala.

Normative data for the original black and white drawings of Snodgrass & Vanderwart (S&V) [2] and the subsequent coloured version [3] have been obtained for several other languages (e.g., Persian, [4]; Turkish, [20]; for more see Table 1). To date, however, there have been few attempts to develop norms for Indian languages, although there are more than 1 billion speakers worldwide. A 2007 study with adult Malayalam speakers to gather norms for the black and white S & V (1980) pictures [26] reported that only 13/40 items were reliably identified by name. They also found low familiarity for the black and white items, which the authors interpreted as a reflection of the age (92/200 over 65), rural background and low level of education (87/200, 0–4 years) of the majority of the participants. In a more recent study in Hindi [14], the most spoken Indian language, 59 Hindi-English bilingual students rated 158 coloured S&V [3] pictures in Hindi for image agreement, name agreement, familiarity, visual complexity, and age of acquisition. A further 40 different Hindi-English bilinguals named the items in Hindi for reaction time measures. Among the rated variables and computed word frequency and syllable length included, only four psycholinguistic variables (image agreement, name agreement, familiarity, and age of acquisition) predicted naming latency.

**Table 1:** Summary of norming studies carried out in different languages.

Norms provided for black and White line drawings of S & V (1980)	Norms provided for coloured version of S & V pictures by Rossion & Pourtois (2004)
British English [9]	Mandarin Chinese [18]
French [27]	Modern Greek [28]
Spanish [24]	Russian [15]
Italian [29]	Persian [4]
Icelandic [30]	Turkish [20]
Chinese [31]	Hindi [14]
American & Chinese [32]	
Japanese [33]	
Canadian French [34]	
Mandarin Chinese [17]	

Regarding other Indian languages, one study has been conducted in Kannada. The study involved 5–16-year-old (n = 100) children providing Kannada norms for 260 Snodgrass and Vanderwart line drawings for one psycholinguistic variable: name agreement [35]. They found high name agreement for 197/260 S&V [2] pictures. The poor name agreement for the remaining 63 items was due to items being identified as ambiguous, more than one specific name, or items elicited English names. Generalizing to adult speakers of Kannada is not straightforward, as name agreement scores have been shown to vary between children and adults [36]. Additionally, the study is limited by only reporting name agreement rather than the other important psycholinguistic variables (i.e., familiarity, visual complexity, image agreement, word frequency and age of acquisition) which influence picture naming in other languages [4, 6, 8, 14, 20].

Given the prevalence of Indian language speakers and growing evidence from other languages studies (Table 1) that normative values are not directly transferable from US-English, this study set out to develop norms for use with younger and older Kannada speakers. In Karnataka, the home state of Kannada, levels of bilingual (40%) and multilingual (13%) speakers are substantially above the national average, with English the most common second language. This provides an opportunity to explore the impact and infiltration of English words and items in the majority language—Kannada—which can inform normative studies in other Indian languages. Following the model of Snodgrass & Vanderwart [2] we set out to collect ratings in Kannada for image agreement, picture-name agreement, familiarity, visual complexity, AoA for 180 R&P [3] colour pictures. The second aim was to validate the relationship between rated psycholinguistic variables, obtained word frequency data and picture-naming latencies in native Kannada speakers who are late bilinguals (i.e., exposed to second language after joining to school, age >7) with English as their second language. The key purpose of generating Kannada norms is to encourage the practical use of pictures in psycholinguistic, cognitive, and clinical research. Acknowledging and understanding the exposure to two languages and where one language is preferred over the other, is an important element of developing future materials. To add to the growing body of evidence regarding similarities and differences across different languages [e.g., 4, 20, 28], we also conduct cross-linguistic comparisons of the psycholinguistic variables between Kannada norms and other languages that have used the R&P coloured pictures [3] and with the original S&V study [2] of black and white line drawings.

## Materials and method

In this study, 185 of the 260 colourized S&V pictures from Rossion & Pourtois [3] were selected. Item selection was polled by the author and four volunteer participants (2 young adults [YA] and 2 older adults [OA]). All five were bilingual speakers with Kannada as their native language and learnt English after the age of 7 in YA and age 12 in OA. These participants were first asked to determine (1) if the pictured items were culturally familiar; and (2) had an equivalent Kannada name. We excluded 75 pictures in total, among them 50 were not culturally familiar and 25 pictures did not have an equivalent name in Kannada language or cognates (i.e., ‘*artichoke*’, ‘*cigarette*’, ‘*accordion*’, ‘*stapler*’, ‘*penguin*’, etc.). Items referred to by their English “loan-names” (now a routine part of Kannada vocabulary, e.g., ‘*shirt*’, ‘*belt*’, ‘*sweater*’, ‘*helmet*’) were retained because these items had equivalent Kannada names.

From the final set of 185 items, five—‘*sun*’, ‘*tiger*’, ‘*scissors’*, ‘*book*’, and ‘*ring*’—were chosen for practice trials as these items had a single dominant name and were familiar to the participants. The remaining 180 items were divided randomly into two sets of 90 items. Participants were randomly assigned to rate one set of 90 items and name the other set of 90. Thus, each of the 180 items was rated by 34 participants and named by the other 34 participants. Word frequency data for the S&V pictures in Kannada were obtained from Sketch engine and included in the analysis.

Seventy-five participants were recruited to participate in the study. Each completed the Montreal Cognitive Assessment (MoCA) [37] Kannada version. Seven of these were excluded following cognitive screening using the MoCA. The remaining 68 participants were recruited in two age bands: 35 aged 19–30 years old (22 females and 13 males), and 33 aged between 60–80-year-old (14 females and 19 males). This was to inform future use of the norms in clinical tools, particularly for older adults.

Each participant completed the Modified Language Proficiency Questionnaire (MLPQ; see S1 Appendix). Twelve of the 68 participants were bilinguals, and the rest were multilinguals. All were native Kannada (L1) speakers of whom 66 had English (L2) as their second language and Hindi as their third language. The other two participants spoke a different second language and had English as their third language. All participants were late bi-/multilinguals, which means they were exposed to one language at birth (Kannada) and to another language or languages (English and/or Hindi) later in childhood or adulthood [38]. English was acquired later among the OA group (12+ years) than the YA group (7+ years).

### Procedure

Data were collected individually in two settings. Twenty of the YA participated at Jagadguru Sri Shivarathreeshwara Institute of Speech and Hearing Mysuru. The other 15 younger participants who were not part of JSS Institute and all of the older adults participated in their own homes. The data were collected over two sessions on different days for each participant. In the first session, after the participant provided informed consent, they completed the Montreal Cognitive assessment MoCA [37], Kannada version. The MLPQ (see S1 Appendix) was administered for demographic purposes and was not included in further analysis (see Table 2). In the second session, participants completed the rating tasks for one set of 90 pictures, and the confrontation naming task for the other 90 items. Therefore, no participant named the same items that they rated, and this was counterbalanced to ensure that each item was named and rated by half of each group. Five-minute breaks were offered within the session following naming or rating 45 items. The overall session length varied between 90–120 minutes in total for YA and up to 150 minutes for OA.

**Table 2:** Demographic data.

Parameters	Groups	
	Younger (35) *M (*SD*)*	Older (33) *M (*SD*)*
Age	23.77 (3.71)	67.2 (4.44)
MoCA scores (/30)	28.17 (1.2)	27 (0.93)
MLPQ—Overall L1 proficiency (100)	82.37 (8.17)	92.48 (4.56)
MLPQ—Overall L2 proficiency (100)	74.97 (12.63)	57.75 (12.84)

*Note*. MoCA: Montreal Cognitive Assessment; MLPQ: Modified Language Proficiency Questionnaire; L1 (Kannada); L2 (English).

### Ethical procedure

The study received ethical approval from the School of Psychology and Clinical Language Sciences Research Ethics Committee at the University of Reading (2019-072-AA). The information sheet was provided in Kannada to each participant, and they were given the opportunity to ask questions about what the study would involve. They were also informed that they were free to withdraw from the study at any point in time without giving any reason. Following this, they were asked to provide their written consent.

### Task design

In the present study, both the tasks and order of completion, were designed to collect data from the same participants for: IA, PNA, FAM, VC, AoA and naming. Name agreement scores were calculated from confrontation naming. Although normative and confrontation naming was conducted on the same day, the stimuli used for both were different.

#### Rating tasks

Each rating task used a Likert rating scale from 1–5 (see S2 Appendix for rating scale). All participants started with rating IA and PNA followed by FAM, VC and AoA together. The order of presentation of pictures was pseudorandomized by creating four sets of the same 90 pictures, which were randomly presented across participants. Therefore, all participants started rating IA and PNA with one set followed by FAM, VC and AOA with another set of the same 90 items in a different order. It was necessary for the PNA rating to follow the IA rating as the participants had to be exposed to the lexical item for both these variables.

*Image Agreement (IA) and Picture Name Agreement (PNA)*. To rate IA, the item name in Kannada was displayed to the participant on a white screen using Microsoft PowerPoint for three seconds in black font Nirmala UI, size ‘72’. This was followed by a blank screen which appeared for five seconds, during which time the participant was required to imagine an image corresponding to the word. Later after 5 secs, they were shown an image representing an item. The participants were then asked to rate how good was the match between their imagination and the displayed image on the Likert scale (1–5). For IA, 5 represents a good match between the name and image they had in mind, with numbers closer to 1 representing a poor match. If participants were unable to mentally imagine a visual image within 5 seconds, they were asked to report ‘cannot imagine’ (CI) for that item.

Following IA, participants rated PNA on how good/poor the match was between the name and picture on the same 1–5 scale. If they knew the item when they saw it but not the name that had been presented, they were asked to write ‘do not know name’ (DKN) for that item. For any items that participant did not recognize, they were asked to report ‘do not know the object’ (DKO). To avoid bias in accepting the given name, and to elicit widest and most common naming responses for the pictured items, participants could also write down their most common response in an extra column on the rating scale. This was required for items rated equal to or below 3 on PNA rating.

*Familiarity (FAM)*, *Visual Complexity (VC) and Age of Acquisition (AoA) rating tasks*. In these rating tasks, as each item appeared on the screen, participants were asked to rate it for familiarity, visual complexity, and age of acquisition. As explained above, the 90 items were presented in a different order to the IA and PNA rating tasks. For each item displayed FAM, VC and AoA were rated on three separate Likert scales before moving on to the next picture for all 90 items. **FAM** was rated on a Likert scale based on their experience of hearing, seeing, and using those items (1–5). The FAM ratings were 5 = familiar down to 1 = unfamiliar. Participants were asked to rate familiarity on lifetime experience not on the immediate prior exposure from previous rating task. **VC** ratings were judgements of the overall intricacy of the item displayed based on the shape, colour, and lines drawn. The 5-point Likert scale went from 5 = sufficient details with 1 = insufficient to convey the item. Finally, participants were asked to rate the age at which they learnt about each pictured item. Given that the Kannada-speaking population are largely bi/multilinguals, it was deemed appropriate to obtain conceptual, rather than lexical, acquisition age. In this rating task, each point on the Likert scale represented an age range: 1 = 0–3 years; 2 = 3–6 years; 3 = 6–9 years; 4 = 9–12 years; and 5 = 12 and above years as such higher scores equate to later age of acquisition of a concept.

#### Confrontation naming

In the confrontation naming task, items were presented one at a time using Psychopy software version 3.1.2 [39]. Participants were asked to name each picture as soon as it appeared on the computer screen. The task comprised 3 blocks. The first block consisted of five practice trials to ensure the participant understood the task efficiently. The second and third blocks were the main task and included 45 items each, with a five-minute break provided between each block. Each trial consisted of a 500ms fixation followed by the presentation of the picture item. A 200ms beep was presented simultaneously with the picture stimuli, which acted as the cue for measuring reaction time. The picture remained on the screen for 3000ms followed by a 2000ms blank screen.

Participants were instructed to say the first name which came to mind in the Kannada language as soon as they saw the picture. Specific instructions were given to elucidate any naming failures: “Do not know the object” (DKO) if they do not know the object, “Do not know the name” (DKN) if they know the object but cannot name it; and tip of the tongue (TOT) if they know the name but are unable to retrieve it. Verbal responses were recorded with the Psychopy voice key, and the reaction time measured from the onset of the presentation of the picture/beep stimulus. Reaction time and name response were extracted for each item for all the participants.

In addition to the ratings collected from the participants, word frequency data for 162 items were obtained from Sketch engine Kannada web 2012 (KNWAC12) database (approximate word count: 11 million). Frequency data were log transformed (Log10 (Counts per million) + 1) and included as a predictor of naming latency in a regression analysis alongside the rated variables.

## Results

The overall mean and standard deviation on MoCA and MLPQ scores of the 68 participants are presented in Table 2. The MoCA scores are slightly but not significantly higher for the younger adults and the MLPQ scores indicate high L1 proficiency in both groups with above average proficiency for L2.

Item by item rating and name agreement data can be found in the S3 *Appendix*. The first step in examining the data was to establish whether to keep the data from the younger and older adults separate or to combine into one set. To address this, correlations were run between the YA and OA mean rating scores for each item for all psycholinguistic variables plus the confrontation naming results (see Table 3). There were strong significant positive correlations for all rated psycholinguistic variables and a moderate correlation for naming reaction time, although all were significant at *p* < .01. These results were cross verified using Fischer Z transformation tests which showed no significant difference between the groups for each correlation across the psycholinguistic variables. As such the group data were then combined for further to provide descriptive summary, cross-linguistic correlations, and regression analyses.

**Table 3:** Correlations between younger and older adults.

Psycholinguistic variables	*r* value
Image agreement	0.662**
Picture name agreement	0.725**
Familiarity	0.715**
Visual complexity	0.585**
Age of acquisition of concept	0.735**
Hstats (confrontation naming)	0.767**
Hpercent (confrontation naming)	0.655**
Reaction time (ms)	0.453**

*Note*^. **^Correlation is significant at *P*>.01 level.

The results of the rating tasks, name agreement and reaction time for the 180 items are contained in Table 4. The mean scores are high for IA and PNA, indicating that the Kannada translation was representative of the image and vice-versa. Similarly, higher FAM and VC mean scores representative of the concepts were familiar and there was sufficient detail to identify the concepts. Low mean rating scores were obtained on average for AoA indicating the concepts were acquired early in life (the majority before 6 years). The results of the rating tasks are explored separately before examining their combined influence on naming latency.

**Table 4:** Descriptive statistics of rating and confrontation naming task.

	N	Mean	*SD*	Range	Median	Skewness
IA (/5)	180	4.51	.61	2.06–5.00	4.76	-2.16
PNA (/5)	180	4.52	.78	1.48–5.00	4.94	-1.82
FAM (/5)	180	4.85	.21	3.73–5.00	4.94	-2.91
VC (/5)	180	4.64	.21	3.76–4.94	4.71	-1.34
AOA (/5)	180	2.54	.70	1.26–4.18	2.50	.20
Hstats	180	.66	.58	.00–2.21	.59	.53
Hpercent	180	.64	.31	.00–1.00	.76	-.58
RT (ms)	176	1634.78	312.96	816.50–2558.40	1635.65	.22

*Note*. IA: image agreement; PNA: picture-name agreement; FAM: familiarity; VC: visual complexity; AOA: Age of acquisition; RT: Reaction Time.

### 1. Rating tasks

A small proportion of items received lower ratings (3 or below) in the rated psycholinguistic variables. On the IA task, 5.77% (40 items—*barrel*, *barn*, *beetle*, *blouse*, *cannon*, *chain*, *cloth pin*, *doorknob*, *desk*, *dresser*, *envelope*, *football*, *football helmet*, *frying pan*, *grasshopper*, *lightbulb*, *lobster*, *gloves*, *ostrich*, *nail filer*, *pitcher*, *pear*, *rhino*, *ruler*, *rocking chair*, *sweater*, *seahorse*, *seal*, *snail*, *snowman*, *suitcase*, *trumpet*, *mushroom*, *spinning wheel*, *vinyl*, *vest*, *vase*, *wagon*, *watering can*, *zebra*) of total responses were those that at least one participant could not imagine (CI response) from the given Kannada name.

In picture-name agreement, the same items resulted in DKN response meaning given item names in Kannada being unknown to at least for one person and consisted of 3.30% of total responses. This suggests that in the image agreement task, the majority of “cannot imagine” responses were due to the Kannada name being unfamiliar rather than a lack of knowledge of the item. However, there were also “do not know the object” responses for10 items—*barrel*, *beetle*, *football*, *lobster*, *nail filer*, *ostrich*, *pear*, *seal*, *seahorse*, *snowman*, indicating that these 10 items were unfamiliar to at least one participant (1.05% of total responses). In addition to these, a further 23 items (*barrel*, *beetle*, *couch*, *football*, *fly*, *doorknob*, *lobster*, *nail filer*, *ostrich*, *pear*, *rhino*, *saw*, *seal*, *seahorse*, *snowman*, *stove*, *spinning wheel*, *vinyl*, *violin*, *watering can*, *wagon*, *zebra)* were rated as having low familiarity by at least one participant (3.6% of total responses), indicating low exposure to these items in daily life.

Regarding VC, 43/180 items (*apple*, *arrow*, *ball*, *barn*, *barrel*, *beetle*, *boot*, *bowl*, *broom*, *comb*, *doorknob*, *dresser*, *eye*, *fly*, *football*, *fox*, *hair*, *garbage can*, *ladder*, *lamp*, *leaf*, *leopard*, *lion*, *lobster*, *kettle*, *knife*, *monkey*, *necklace*, *nail filer*, *ostrich*, *pear*, *rhino*, *sailboat*, *seahorse*, *snowman*, *stove*, *seal*, *spinning wheel*, *vest*, *violin*, *vinyl*, *whistle and wagon*) were regarded by at least one participant as having insufficient visual detail (7.65% of total responses) to identify the item. AoA ratings revealed that 105 items, accounting for nearly 60% of the concepts were learnt earlier in life before the age of 6 years.

**Fig 1:**
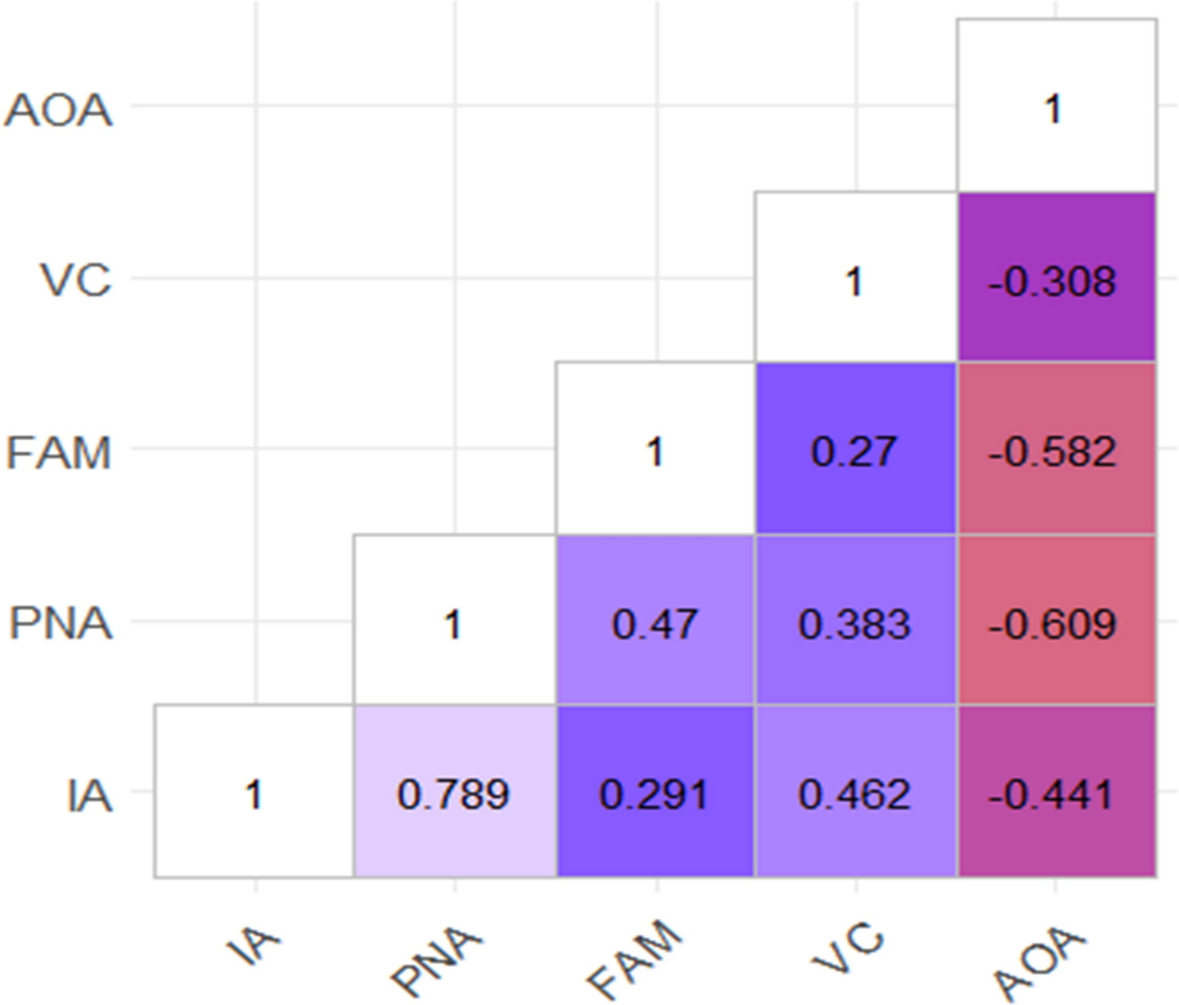
Correlation matrix of rated psycholinguistic variables.

Spearman’s correlations were used to evaluate the relationship between the rated variables (Fig 1). There were strong to moderate significant correlations between the majority of variables with the exception of familiarity with both image agreement (*r* = 0.291) and visual complexity (*r* = 0.27), which produced significant but weak correlations. These results mirror what has been reported in other studies.

### 2. Confrontation naming

Name agreement in the form of the *H*stats was calculated from confrontation naming data for each item (Table 4). *H*stats differs from *H*percent by considering all the naming responses given for an item even if it is not named in Kannada. On the other hand, *H*percent is the percentage of name agreement obtained for each item by calculating only the total number of correct responses (items correctly named in Kannada) provided by the total number of participants multiplied into 100 (see Table 4 for summary statistics). Reaction time measures for four items (*Barn*, *Football*, *Lobster*, *and Sweater*) were removed from the confrontation naming latency as these items had zero correct responses, accounting for 2.2% of the 180 items RT.

The *H*stats scores for the 180 items demonstrated a range of name agreement from complete to very low name agreement across items. Forty-two items (24%) had *H*stats of zero, meaning they had complete name agreement. Twenty of the 180 items had *H*stats score above 1.5 indicating very low name agreement (*arrow*, *barrel*, *bed*, *bird*, *boot*, *broom*, *cat*, *clothes pin*, *dress*, *iron*, *knife*, *owl*, *pear*, *socks*, *stove*, *umbrella*, *vase*, *wagon*, *watering can*, *zebra*) with multiple alternative naming responses. The remaining 118/180 items represent *H*stats score below 1.5 suggesting more than one name was being used by participants to represent these items.

Confrontation naming data were analysed for reaction time (ms) and accuracy. Ten different types of naming responses were observed, (1) Correct items produced as the Kannada translation of the English item or a synonymous Kannada word = 63.6% of responses; (2) Naming failure (no response e.g., tip of the tongue or unknown item or unknown item name) = 17.69%. (3) Translation (correct English names) = 8%; (4) Semantic error: semantic substitution e.g., another item from the same semantic category = 4%; (5) Alternate names: semantically acceptable related Kannada names = 2%; (6) Superordinate error (name of the category of the target) = 1.92%; (7) Visual error (visually related item) = 2.1%; (8) Incomplete (partial name produced e.g., first or second half only) = 0.31%; (9) Description of the item = 0.19%; and (10) Unrelated (item not semantically or visually related to the target) = 0.19%.

To calculate the mean reaction time for each item name, incorrect responses and naming failures were first removed, which accounted for 36% of the responses. Naming latencies more than three standard deviations from the mean were excluded to reduce the effect of outliers which resulted in 1.13% of total responses. Naming latencies for all 176 items are reported in S3 *Appendix*. The overall mean naming latency for 176 coloured S&V pictures was 1634.78ms (*SD* = 313.78). Further, only correct responses were considered for the regression analysis and cross-linguistic correlations.

#### Predictors of naming latency

Simultaneous multiple linear regression analysis was conducted to validate the relationship between rated psycholinguistic variables and obtained word frequency on naming latencies. The dependent variable was mean RT (ms) and independent predictor variables were IA, PNA, FAM, VC, AoA, *H*stats, *H*percent and word frequency. PNA and *H*percent were removed from the final model as these variables were outside of multicollinearity tolerances: PNA (VIF = 4.75, PNA vs. IA r = 0.789); *H*percent (Condition Index = 143, *H*percent vs. *H*stats r = -0.814). The above psycholinguistic variables predicted naming latency in Kannada yielded an adjusted *R*^*2*^ = 0.638, *F*_(6,156)_ = 46.77, and *p* < .001 (Table 5).

**Table 5:** Standardized coefficients and *R*^*2*^ changes in multiple regression analysis.

Variable(s)	Beta	*t*	*p*	VIF
**IA**	-.133	-2.24	.026	1.51
**FAM**	.189	2.99	.003	1.71
**VC**	-.028	-.53	.591	1.19
**AOA**	.389	5.57	< .001	2.09
**Hstats**	.556	8.96	< .001	1.65
**Word fq**	.013	0.22	.820	1.34

*Note*. IA: image agreement, FAM: familiarity, VC: visual complexity, AOA: Age of acquisition of concept, and Hstats: name agreement scores, Word fq: Word frequency.

Four variables–IA, AoA, *H*stats and FAM were significant predictors of naming latency, while VC and word frequency were not (Table 5). The most robust predictor was name agreement (*H*stats; *b* = .556, *t* = 8.96, *SE* = 31.74, *p* < .001) which suggests that reaction time is faster for items with greater name agreement. AoA of concept was also a strong predictor (*b* = .389, *t* = 5.57, *SE* = 31.20, *p* < .001) indicating faster reaction time for concepts acquired earlier in life. Items with higher image agreement (IA) scores were named more quickly (*b* = -.133, *t* = -2.24, *SE* = 30.80, *p* < .01). Of the four significant variables, item familiarity did not go in the expected direction; latency was slower for more familiar objects (*b* = .189, *t* = 2.99, *SE* = 119.07, *p* < .001). This unexpected change in beta sign in the regression analysis is possibly due to positive net suppression [40]. This occurs when one predictor variable is more strongly correlated with other predictor variables and these other variables are more strongly correlated with the dependent variable. In the current study familiarity may have acted as a suppressor variable in the regression model as it showed a weak relationship with naming RT (r = 0.28) but a strong relationship with AoA (r = 0.58) and a moderate relationship with *H*stats (r = 0.43). In comparison, AoA and *H*stats both demonstrated a strong relationship with naming RT (r = 0.54 and r = 0.68, respectively). Neither visual complexity (*b* = -.028, *t* = -.539, *SE* = 81.38, *p* > .05) nor word frequency (*b* = .013, *t* = .22, *SE* = 32.95, *p* > 0.05) resulted as significant predictors of naming latency in Kannada.

#### Cross-linguistic correlations

Kannada results from rating and confrontation naming tasks were compared to previous norming studies in other language using S&V (black and white or coloured) picture stimuli (Table 6). Results ranged from weak, non-significant correlations to strong, significant correlations (*r* = -.04 to *r* = .673). AoA, demonstrated the most consistent and strong relationship with other languages and visual complexity the weakest. Kannada norms showed the strongest relationship to Persian, Turkish and Hindi and only a limited relationship with English and French.

**Table 6:** Comparison of psycholinguistic variables of present study with original black and white line drawing and similar studies which have used colourised version of Snodgrass and Vanderwart pictures.

Variables (N)	S&V (1980) 179	R&P (2004) 180	Weekes et al., (2007) 163	Dimitropoulou et al., (2009) 180	Tsaparina et al., (2011) 179	Bakhtiar et al., (2013) 139	Raman et al., (2014) 179	Ramanujan et.al., (2020) 130
**Languages**	**American English**	**Belgian French**	**Mandarian Chinese**	**Modern Greek**	**Russian**	**Persian**	**Turkish**	**Hindi**
**IA**	.209**	.221**	.120	NA	.257**	.412**	.334**	.423**
**FAM**	.099	-.167*	.320**	NA	.340**	.257**	.291**	.400**
**VC**	-.040	-.118	-.050	-.146	.228**	-.185*	-.182*	.278**
**AOA**	NA	NA	.655**	.672**	NA	.673**	.671**	NA
**Hstats**	.130	.063	.200*	.216**	.028	.245**	.460**	.210*
**Hpercent**	.078	NA	.286**	.202**	.103	.312**	.512**	.084
**RT (ms)**	NA	.357*	.457*	NA	NA	.482*	NA	NA

*Note*. **Correlation is significant at 0.01 level.

*Correlation is significant at 0.05 level. IA: image agreement; Name agreement: Hstats and Hpercent; FAM: Familiarity; VC:Visual complexity; AOA: Age of acquisition of concept and RT(ms): Reaction time in ms.

## Discussion

This study is the first to provide psycholinguistic norms for coloured Snodgrass & Vanderwart [3] pictures in Kannada from adult Kannada-English bilingual speakers. Ratings were collected for five psycholinguistic variables: image agreement (IA), picture-name agreement (PNA), familiarity (FAM), visual complexity (VC), age of acquisition (AoA) in line with previous normative studies e.g., Mandarin Chinese [18]; Persian [4]; Turkish [20]; Hindi [14]. These normative data are intended to be used in the development of psycholinguistic, cognitive, and clinical experimental paradigms.

The majority of the 180 items were well known to the participants and easily interpretable as indicated by their high ratings for IA, PNA, FAM and VC. However, responses to some items indicate caution is required in selecting items for experimental materials depending on the nature of the task. For example, a proportion of the translated Kannada names were unknown to the participants, in that they could not imagine the object from the translated Kannada name. Such lexical items would not be appropriate for lexical decision, recognition, or comprehension experiments. Similarly, a large proportion of items had more than one commonly produced name in confrontation naming which has implications for the design and interpretation of word production, recognition, and recall tasks. In addition, it is important to consider the limits of the items normed in the current study. The mean ratings were high for all psycholinguistic variables using the 5-point Likert scale (>4.5/5), indicating limited variation within the data. This lack of variation is likely to impact the ability to make experimental manipulations and may reduce power to detect experimental effects. AoA had greater variation, however the Likert scale used was relatively arbitrary in terms of representing age bands.

Despite the removal of culturally inappropriate items, only 64% of confrontation naming responses were classed as correct. Items that were rated below 3/5 for IA, PNA, FAM and VC were more likely to elicit incorrect responses in the confrontation naming task. The majority of non-correct responses (36%) were naming failures (i.e. cannot produce the name which includes DKN, DKO and tip of the tongue responses), translation error (English name produced) or semantically related responses. Naming failures of items in confrontation naming were not surprising, given that in the PNA task, 40 items were rated as unknown, or the Kannada name was unknown by at least one participant. The percentage of naming failures were relatively higher in confrontation naming (17.9%) compared to picture-name agreement rating (4.35%) and this highlights the nature of task involved. The presence of naming failures in both tasks suggests that not all participants are equally exposed to all the items. The naming errors resulted from participants not knowing the Kannada names for pictured items or using the more familiar English names. The participants in the study were bilinguals who are used to switching between languages, and so may be used to using the English rather than Kannada names for some items. However, the present study emphasizes that items which are present in both language and cultural environment may still elicit naming failures (e.g., *barn*, *nail filer*, *broom*, *blouse*, *garbage can etc*.) as these are taken from American English and drawn according to Western style and slight changes in the picture according to Indian environment might result in better rating and naming responses. Nevertheless, a definitive explanation of the linguistic and/or cultural source of naming failures requires further experimental testing. Hence, we suggest that based on these norms, stimuli selection should pay careful attention to the psycholinguistic variables and naming latency depending on the task at hand. Further, these norms are helpful for future decisions of whether to accept certain responses as acceptable or not in Kannada-English bilinguals. For example, in speech production or comprehension assessments.

In the present study, greater image agreement and name agreement (*H*stats) and younger age of concept acquisition predicted faster naming latencies. Visual complexity of the coloured pictures and word frequency were not significant predictors (Table 5). Together these variables, with familiarity, accounted for 62.3% of variance in explaining naming latency. These findings replicate previous psycholinguistic research on word retrieval e.g., in French [8], Mandarian Chinese [18], Persian [4], Hindi [14] indicating that the psycholinguistic ratings and name agreement statistics produced for Kannada-English bilinguals in the current study are reliable. However, there is a degree of dialectal variation within Kannada speaking population across Karnataka and, therefore, some caution might be taken in applying these norms to populations outside of the Southwest part of Karnataka where the data were collected.

As noted by Alario et.al., [8] AOA and NA are reported to have independent effects on naming latency. In the current study with adult Kannada speakers, NA was the most robust predictor of naming latency followed by AOA. Alario et al., [8] proposed that name agreement (*H*stats) reflects the stage of lexical retrieval involving accessing the spoken word. It is generally assumed that an object with more than one possible name will slow down the naming response because of competition between activated words [10, 14]. In the present study because the participants were bilinguals, they need to suppress multiple activated words as well as selecting the correct language. Hence, name agreement may have a relatively stronger influence on naming latency and our study supports another recent study in Hindi-English bilinguals on name agreement [14].

Familiarity had an influence on naming latency but unexpectedly, in the regression analysis the coefficient of familiarity was positive. This could have resulted in familiarity supressing the error variance of variables AoA and *H*stats more than predicting the variance of naming latency. Another possible reason for the positive correlation may be due to high rated familiarity. Despite clear instruction on rating familiarity, there may possibly have been an influence on rating the low frequency items as familiar based on the previous exposure to the items in the image agreement rating. The influence of FAM on naming latency is inconsistent across studies [4, 8]. The present study is in line with Spanish [16]; Mandarin Chinese [18]; and Hindi [14] studies which depicted influence of familiarity of naming latency but opposite to predicted direction.

We also found significant effects of image agreement on confrontation naming latencies, and this is well-matched with the results of previous studies using black and white line drawings in American English [2], French [5], Spanish [16] and French [8]. In contrast, a normative study on Mandarin Chinese [18] reported IA to be a non-significant predictor based on the quality of the images. However, more recent studies using coloured S&V pictures in Persian [4] and Hindi [14] have found IA to be significant predictor on naming latency. Although image agreement depends on the quality of the image per se, cultural effects also play role in determining the effects on naming latency. For example, within the present study we noticed the item name ‘*lamp*’ in Kannada elicited a quite different mental representation than the object depicted in the coloured S&V stimuli. This highlights the importance of norming items within languages.

In the present study, visual complexity had no significant effects on naming latency in Kannada, which is compatible with recent studies using similar coloured S&V pictures (e.g., Mandarin Chinese [18] Persian [4] and Hindi [14]). The cross-linguistic comparison of Kannada norms with other studies showed least compatibility for visual complexity. This suggests that details required for visual object recognition is not very sensitive in predicting speed of naming for coloured S&V pictures.

Finally, word frequencies were not significant predictors of naming latency in Kannada. This is in line with previous studies in French [8], Mandarin Chinese [18], and Hindi [14]. It is challenging to include obtained word frequency data from a corpus as a variable where there is large discrepancy for items with low name agreement.

Our main purpose in this study was to establish Kannada norms for the coloured S&V stimuli. Cross-linguistic comparisons were conducted to compare the Kannada normative data with data from seven different language studies who had used coloured S&V pictures [3] and with original S&V [2] study (Table 6). It is important to note that cross-linguistic correlations emphasize the significance of having language specific norms. The correlation strength varied from very low to moderate to high level between *r* = -0.04 to *r* = 0.673. It is evident that the Kannada norms collected here closely resemble those in Persian [4] on all the variables (IA, FAM, VC, AOA, *H*stats, *H*percent and RT) and Turkish [20] expect for RT. There was also close correspondence with Mandarin Chinese [18] with the exception of two variables (IA and VC) and Hindi [14] with the exception of *H*stats. The remaining studies (e.g., [2, 3]) correlated weakly but significantly with the Kannada norms for one or two variables. Overall, AOA was the only variable that correlated significantly and was consistently sensitive across studies. In the present study AOA was measured in terms of concept acquisition rather than word learnt [4, 18, 20, 28] that the age at which concepts are learnt is similar to the age at which the object names are also learnt. Variable cross-linguistic correlations are not surprising given that the familiarity and frequency of the stimuli vary across culture and linguistic environment [4, 41, 42].

## Conclusion

In summary, this is the first study to report norms for 180 Snodgrass & Vanderwart colorized pictures provided by Rossion & Pourtois [3] in Kannada from adults. Normative data were provided for six psycholinguistic variables: image agreement, picture-name agreement, familiarity, visual complexity, age of acquisition of concept and name agreement measures (Hstats). Confrontation naming latencies were predicted by the psycholinguistic variables. Cross-linguistic comparisons highlighted the need for developing such norms for different language and how exposure to S&V pictured items differs across cultures and linguistic environment. The norms produced in the present study are reliable and can be used to produce experimental paradigms and clinical assessments in Kannada-English bilinguals.

The normative data can be found as S3 Appendix.

## Supporting information

S1 Appendix MLPQ modified language proficiency questionnaire. (PDF)

S2 Appendix Rating scale. (PDF)

S3 Appendix (XLSX)

S1 Fig Correlation matrix of rated psycholinguistic variables. *Note*. Correlation ranges (-1 to +1). IA: image agreement; PNA: picture-name agreement; FAM: Familiarity; VC:Visual complexity; AOA: Age of acquisition of concept.
(TIF)
