# Supplementary material for: Standardizing norms for 180 coloured Snodgrass and Vanderwart pictures in Kannada language
Source: PLoS One. 2022 Apr 5;17(4):e0266359. doi: 10.1371/journal.pone.0266359 (PMC8982856; doi:10.1371/journal.pone.0266359)
Supplement: S1 Appendix — (PDF) [file pone.0266359.s001.pdf]

APPENDIX 3

MODIFIED LANGUAGE PROFICIENCY QUESTIONNAIRE

Developed by Yathiraj A., Jain S.N., and Amruthavarshini B. (2018)

Name: Age:

Gender: Male / Female Education:

Occupation:

Instructions: Please read the below given information carefully and choose the most appropriate choice. Respond to all eight points by either filling in blanks or ticking (✓) the most appropriate response. (***Note:*** L1 refers to the first language that you learnt; L2 refers to the second language that you learnt; L3 refers to the third language that you learnt)

****

1. Name all the languages you have learnt since your childhood in the order of acquisition of the language.

**Order of Languages acquired**	**Language Name**
L1	
L2	
L3	

2. Since when have you been using your L1, L2 and L3 for understanding, speaking, reading and writing? *(**Note**. Please tick (✓) one duration per language for understanding, speaking, reading, & writing)*

**Duration in years**	**Understanding**			**Speaking**			**Reading**			**Writing**		
	**L1**	**L2**	**L3**	**L1**	**L2**	**L3**	**L1**	**L2**	**L3**	**L1**	**L2**	**L3**
Less than 5 years												
5 to 10 years												
10.1 to 15 years												
Greater than 15 years												

3. How would you mark your level of proficiency for understanding, speaking, reading, and writing? *(**Note**. Please tick (✓) one level proficiency per language for understanding, speaking, reading, & writing)*

Level of Proficiency	Understanding			Speaking			Reading			Writing		
	L1	L2	L3	L1	L2	L3	L1	L2	L3	L1	L2	L3
Low proficiency												
Fair proficiency												
Good proficiency												
Native like/perfect proficiency												

4. How would you rate your ability to switch between the languages? *(**Note**. Please tick (✓) one of the ratings)*

Rating Scale	Response (✓)
Low Ability	
Fair Ability	
Good Ability	
Perfect Ability	

5. Please tick (✓) which language you use maximum for the below mentioned situations: *(**Note**. Please tick (✓) one language per situation)*

Sl. No.	Situations	L1	L2	L3
a	Interaction with family			
b	Education/ work			
c	Listening to instruction tapes at school			
d	Text books			
e	Dictionary			
f	Story books			
g	Newspapers			
h	Internet source			
i	Writing			
j	Interacting with friends			
k	Interacting with neighbours			
l	Watching TV/ YouTube			
m	Listening to the radio (music)			
n	Market places			

6. On a scale of one to four, how often do you use the languages known to you in the following situations? *(Rating key: 1 = never; 2 = Sometimes; 3 = Most of the time; 4 = Always;* ***Note****. Please write the numbers 1, 2, 3, or 4, for each situation per language).*

Sl. No.	Situations	L1	L2	L3
A	Interaction with family			
B	Schooling/ work			
C	Listening to instruction tapes at school			
D	Text books			
E	Dictionary			
F	Story books			
G	Newspapers			
H	Internet source			
I	Writing			
J	Interacting with friends			
K	Interacting with neighbours			
L	Watching television/ YouTube			
M	Listening to the radio (music)			
N	Market places			

7. How frequently do others identify you as a native speaker based on your accent or pronunciation in the language? *(****Note.*** *Please tick (✓) one rating per language)*

Rating Scale	L1	L2	L3
Never			
Sometimes			
Most of the time			
Always			

8. For how many hours do you use the following languages? *(****Note****. Please tick (✓) one duration per language)*

Duration	L1	L2	L3
Greater than 2 hours			
Greater than 3 hours			
Greater than 4 hours			
Greater than 5 hours			

Note: Refer Scoring key for analysis

APPENDIX 3.1

SCORING KEY

MODIFIED LANGUAGE PROFICIENCY QUESTIONNAIRE

Developed by Yathiraj A., Jain S.N., and Amruthavarshini B. (2018)

Instructions to professional scoring the scale: Please score the responses on a scale of 1 to 4 for each skill / question as directed.

****

1. Name all the languages you have learnt since your childhood in the order of acquisition of the languages.
No score *(Information to be used for descriptive analysis)*

2. Since when have you been using your L1, L2 and L3 for understanding, speaking, reading and writing?

Duration (in years)	Scores	Understanding			Speaking			Reading			Writing		
		L1	L2	L3	L1	L2	L3	L1	L2	L3	L1	L2	L3
Less than 5 yrs	1												
5 to 10 yrs	2												
10.1 to 15 yrs	3												
Greater 15 yrs	4												
**Total Scores**		**L1 = /16**				**L2 = /16**				**L3 = /16**			

3. How would you mark your level of proficiency for understanding, speaking, reading, and writing?

Level of Proficiency	Scores	Understanding			Speaking			Reading			Writing		
		L1	L2	L3	L1	L2	L3	L1	L2	L3	L1	L2	L3
Low proficiency	1												
Fair proficiency	2												
Good proficiency	3												
Native like/perfect proficiency	4												
**Total Scores**		**L1 = /16**				**L2 = /16**				**L3 = /16**			

4. How would you rate your ability to switch between the languages?

**Rating Scale**	**Scores**	**Response**		
Low Ability	**1**			
Fair Ability	**2**			
Good Ability	**3**			
Perfect Ability	**4**			
**Total Scores**		**L1 = /4**	**L2 = /4**	**L3 = /4**

5. Tick (✓) which language you use maximum for the following situations:
No score *(Information to be used for descriptive analysis)*

6. On a scale of one to four, how often do you use the languages known to you in the following situations? *(**Instruction to professional scoring the scale**: Total the ratings given per language).*

**Sl. No.**	**Situations**	**L1**	**L2**	**L3**
a	Interaction with family			
b	Schooling/ work			
c	Listening to instruction tapes at school			
d	Text books			
e	Dictionary			
f	Story books			
g	Newspapers			
h	Internet source			
i	Writing			
j	Interacting with friends			
k	Interacting with neighbors			
l	Watching television/ YouTube			
m	Listening to the radio (music)			
n	Market places			
**Total Score**		**/56**	**/56**	**/56**

7. How frequently others identify you as a native speaker based on your accent or pronunciation in the language?

Rating Scale	Scores	L1	L2	L3
Never	**1**			
Sometimes	**2**			
Most of the time	**3**			
Always	**4**			
**Total Score**		**/4**	**/4**	**/4**

8. For how many hours do you use the following languages?

Duration	Scores	L1	L2	L3
Greater than 2 hours	**1**			
Greater than 3 hours	**2**			
Greater than 4 hours	**3**			
Greater than 5 hours	**4**			
**Total Score**		**/4**	**/4**	**/4**

**L1**	**/100**
**L2**	**/100**
**L3**	**/100**
