# Supplementary material for: Standardizing norms for 180 coloured Snodgrass and Vanderwart pictures in Kannada language
Source: PLoS One. 2022 Apr 5;17(4):e0266359. doi: 10.1371/journal.pone.0266359 (PMC8982856; doi:10.1371/journal.pone.0266359)
Supplement: S2 Appendix — (PDF) [file pone.0266359.s002.pdf]

Appendix-2 [Rating Scale for psycholinguistic variables]

Name: **Age:** **Gender:**
Set:

Rating Scale for Image agreement, Picture-Name agreement

1. **Image Agreement:** Word will appear on the screen, and you need to imagine a picture which resembles the written word for it. Later, an image will appear on screen, and you need to rate for the match between the image you represented mentally, and the image shown on the screen on 5-point rating scale.
   1---------2---------3---------4---------5
   1= Low agreement *(Poor match between the mental representation of your image and the image shown on the screen)*
   5= High Agreement *(Good match between the mental representation of your image and the image shown on the screen)*

2. **Picture-Name Agreement:** Rate how for you agree or don't agree the name for an image shown above.
   1---------2---------3---------4---------5
   1= Low agreement *(Poor match between the word and the picture displayed on the screen)*
   5= High Agreement *(Good match between the image and the word displayed on the screen)*

3. **Alternate most common name:** If you are rating for any picture-name agreement below or equal to '**3**'. Please give the alternate name for that particular image or write DKN (if you don't know the name), DKO (if you don't know the object) or TOT-Tip of the Tongue (if you know the object and name but cannot name it at this moment)

IA	PNA	Alternate most common names

Rating Scale for Familiarity, Visual complexity and Age of acquisition.
Please rate for the following measures on 5 Point rating scale.

1. **Familiarity Rating:** You need to rate for the objects appeared on the screen based on how familiar or how often you come across object in your day-to-day life experience and should not be based on the exposure from previous rating task.
   1---------2---------3---------4---------5

1= Unfamiliar (If you do not know the object displayed on the screen at all)
5= Very Familiar (If you come across the object appeared on the screen quite often)

2. **Visual Complexity Rating:** Please rate the object appear on the screen only based on the quality of image. How simple or the complex the image is? Rating should be purely on the intricacy of the image itself.

   1----------2----------3----------4----------5

   1= Complex (the pictures with less details in terms of colour, shape, and quality of image)
   5= Simple (the pictures which has all the necessary details in terms of colour, shape, and quality of image)

3. **Age of Acquisition:** Please rate for the objects appear on the screen at what age you learnt about the image itself. (Please keep in mind: You need to mention at what age you learnt about that image and not on the age at which you learnt to say the name for the image)

   1= 0-3 years, 2= 3-6 years, 3= 6-9 years, 4= 9-12 years, 5= 12 and above years

**Familiarity Rating**	**Visual Complexity Rating**	**Age of Acquisition Rating**

THANK YOU
