# Supplementary figures and images for: Standardizing norms for 180 coloured Snodgrass and Vanderwart pictures in Kannada language
Source: PLoS One. 2022 Apr 5;17(4):e0266359. doi: 10.1371/journal.pone.0266359 (PMC8982856; doi:10.1371/journal.pone.0266359)

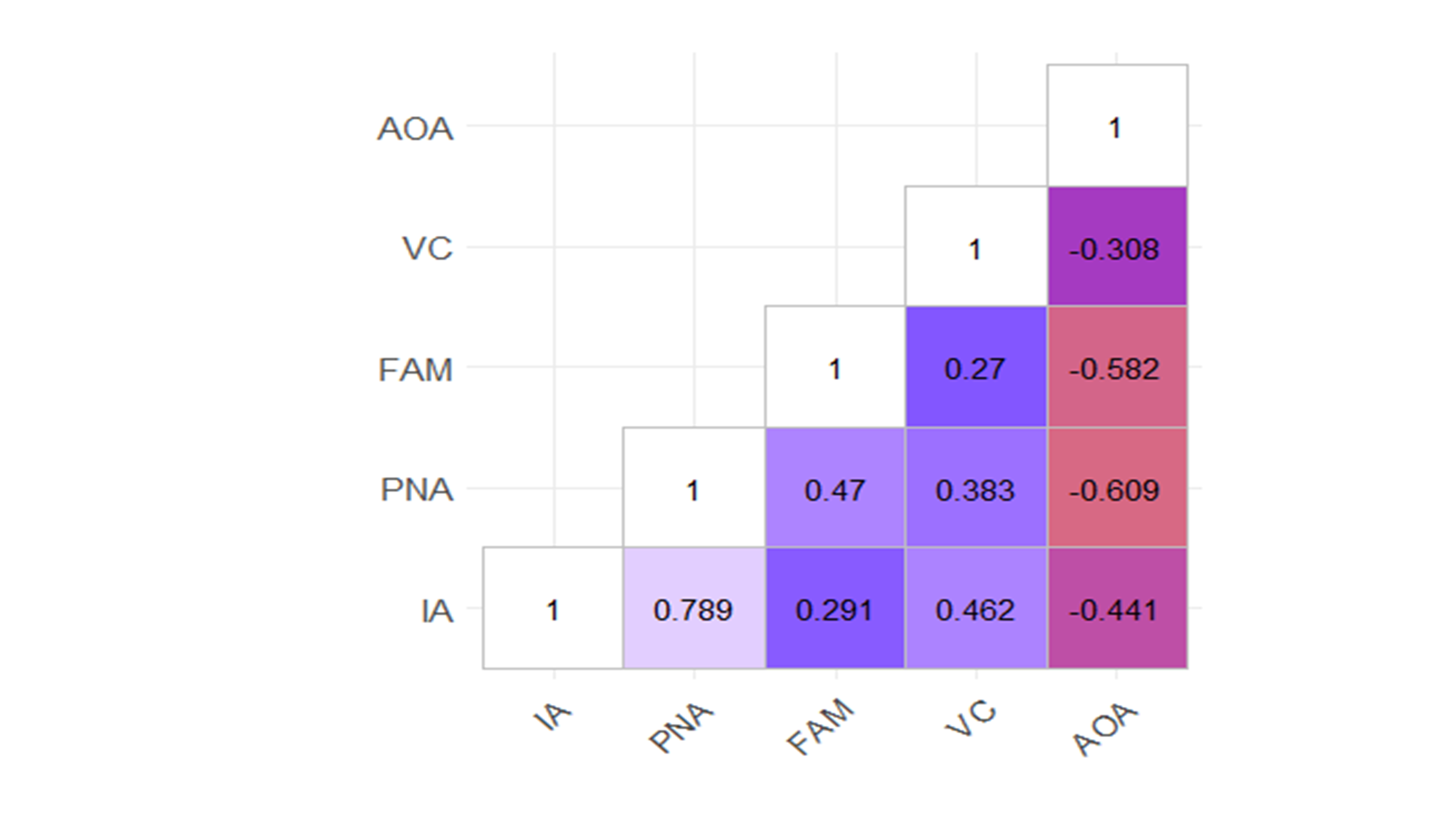

Supplement: S1 Fig — Note. Correlation ranges (-1 to +1). IA: image agreement; PNA: picture-name agreement; FAM: Familiarity; VC:Visual complexity; AOA: Age of acquisition of concept. (TIF) [file pone.0266359.s004.tif]
